# Supplementary material for: Mitochondrial gene editing and allotopic expression unveil the role of orf125 in the induction of male fertility in some Solanum spp. hybrids and in the evolution of the common potato
Source: Plant Biotechnol J. 2025 Mar 22;23(5):1862–75. doi: 10.1111/pbi.70012 (PMC12018842; doi:10.1111/pbi.70012)
Supplement: Supplementary file 1 — Figure S1 Map of somatic hybrid mtDNA. [file PBI-23-1862-s001.docx]

**a.**


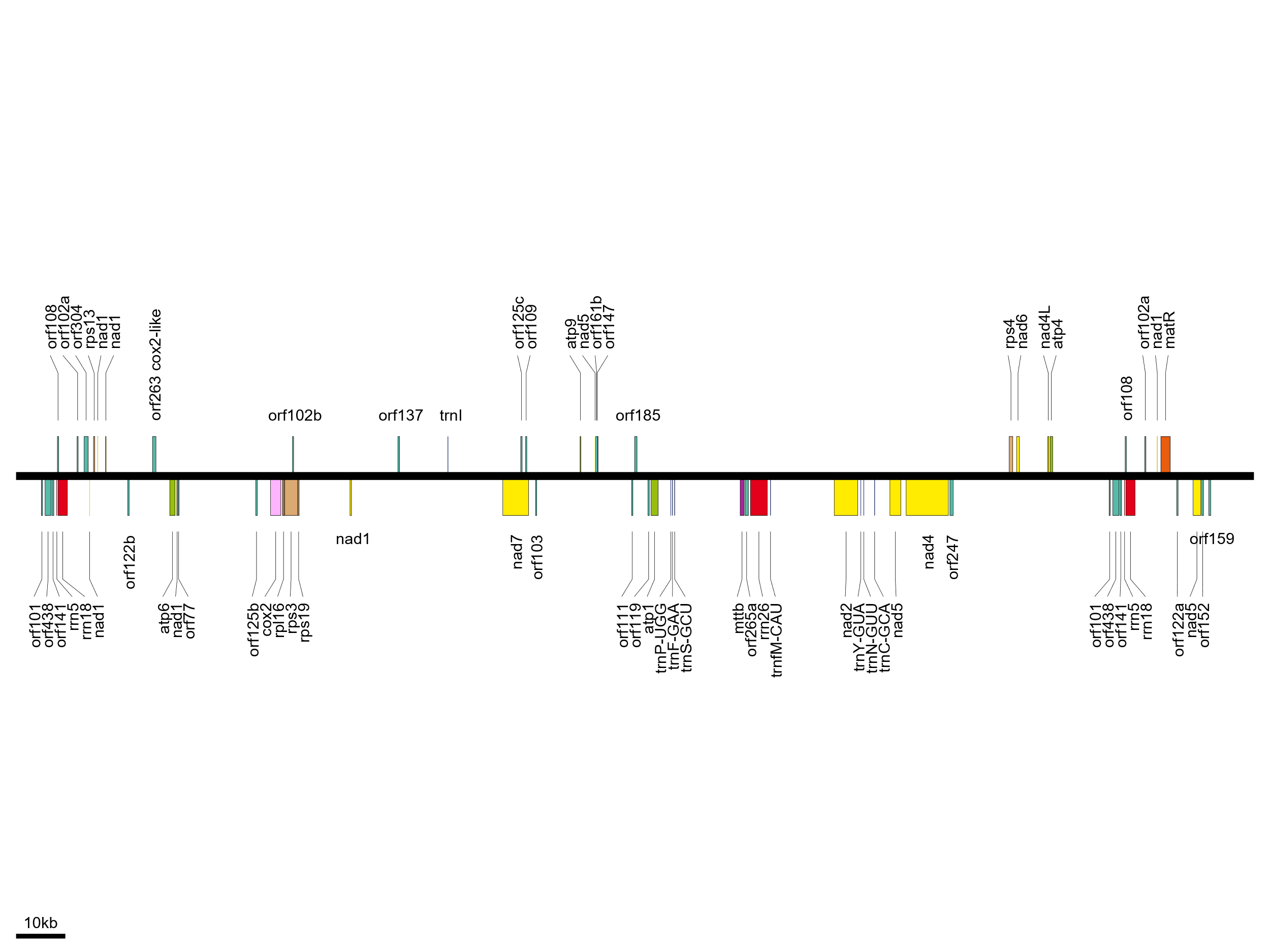


**
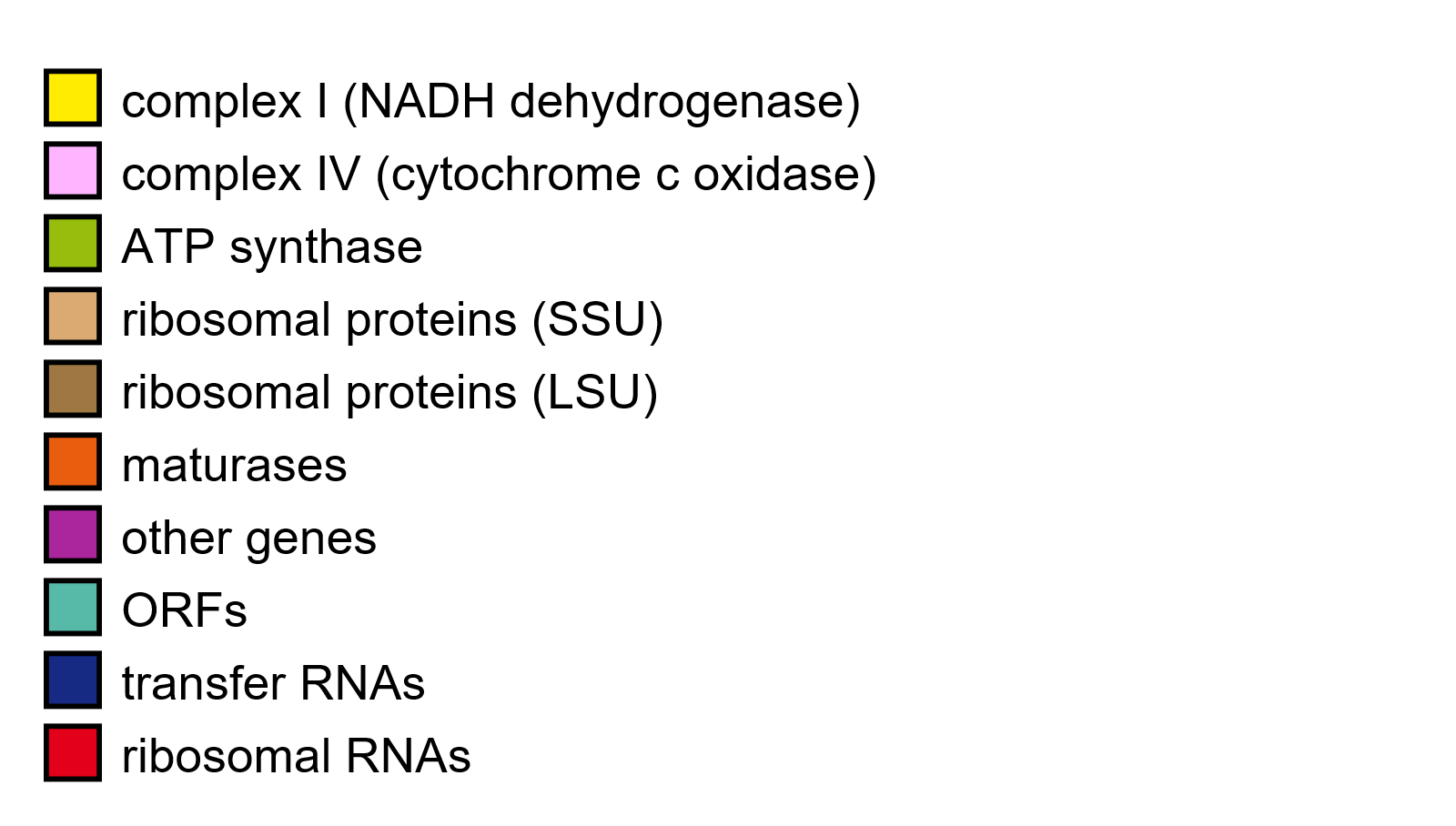
**

**Figure S1**. Map of somatic hybrid mtDNA. **a.** SH9A, chromosome 1, 251363 bp, GenBank acc.no: ON682437.


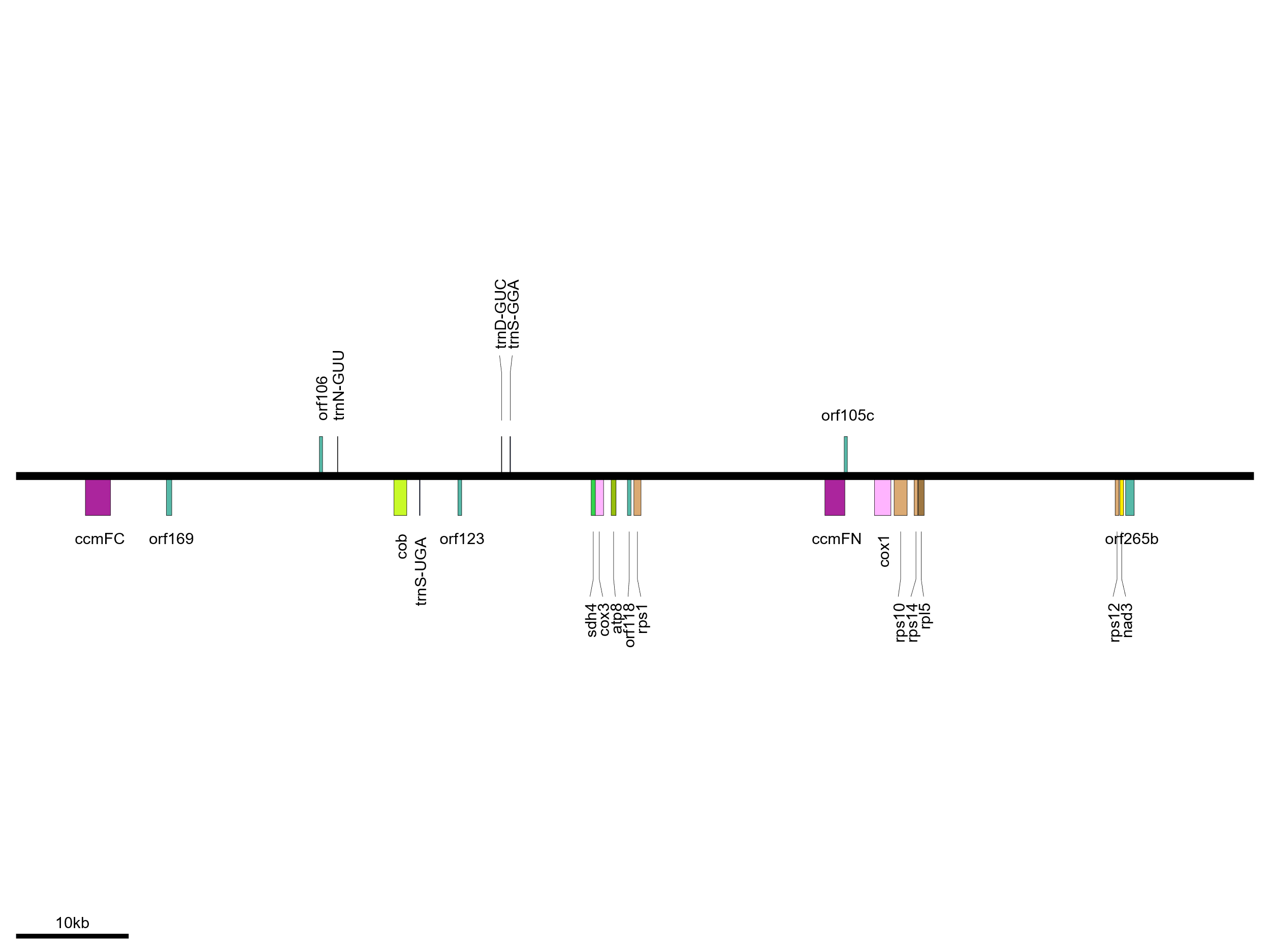
**b.**

**
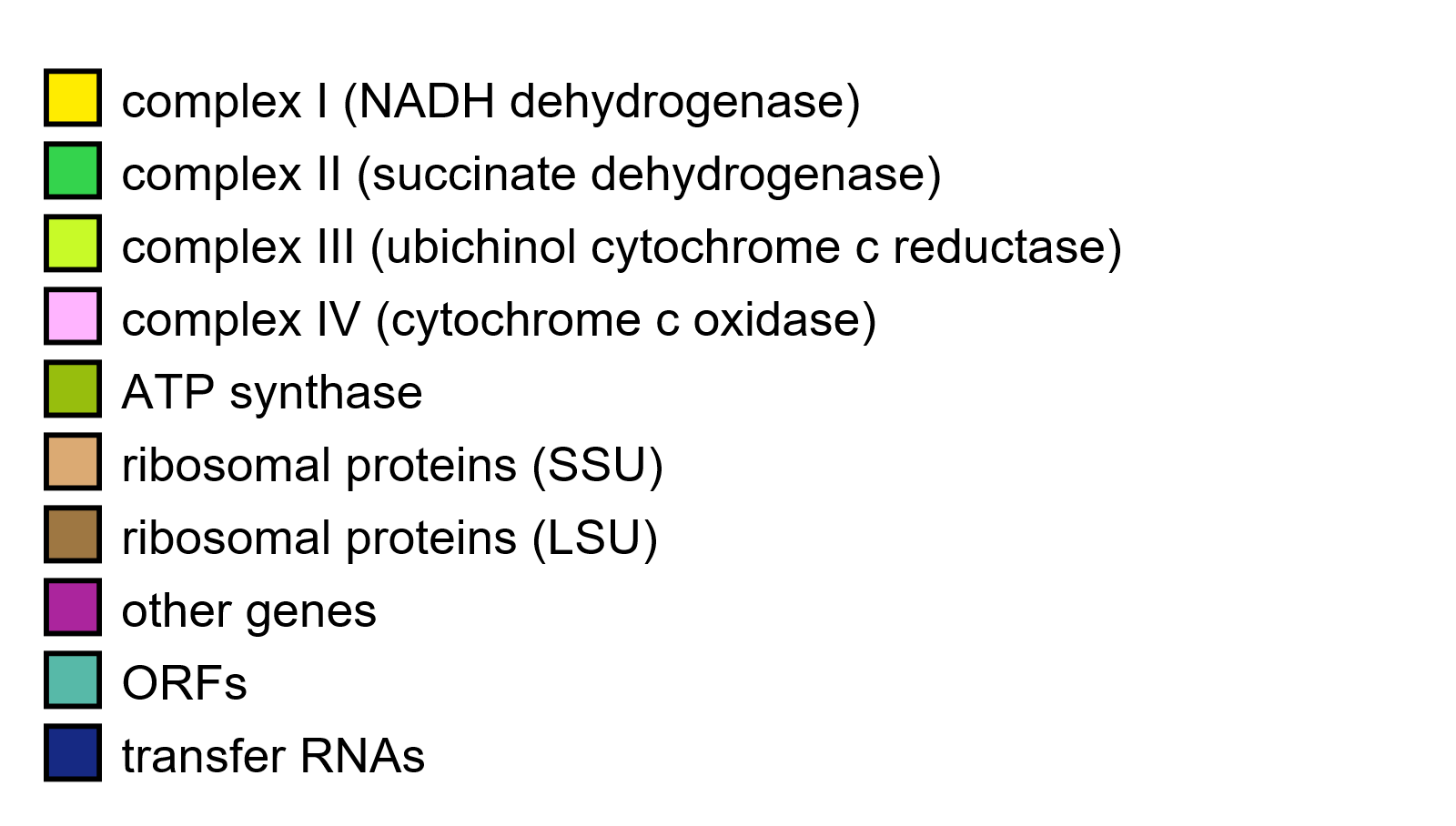
**

**Figure S1**. Map of somatic hybrid mtDNA. **b.** SH9A, chromosome 2, 109928 bp, GenBank acc.no: ON682438.

**c.**


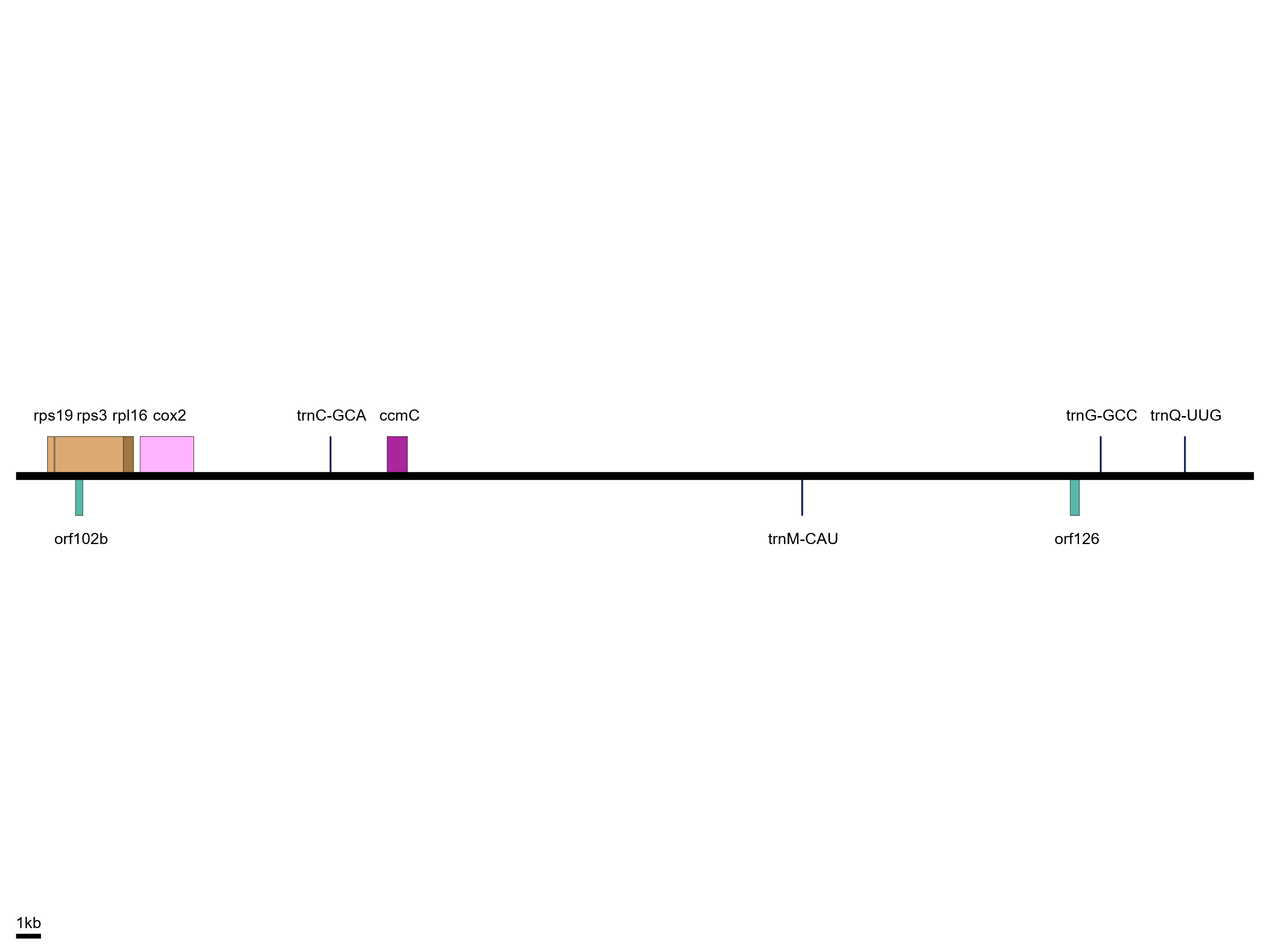


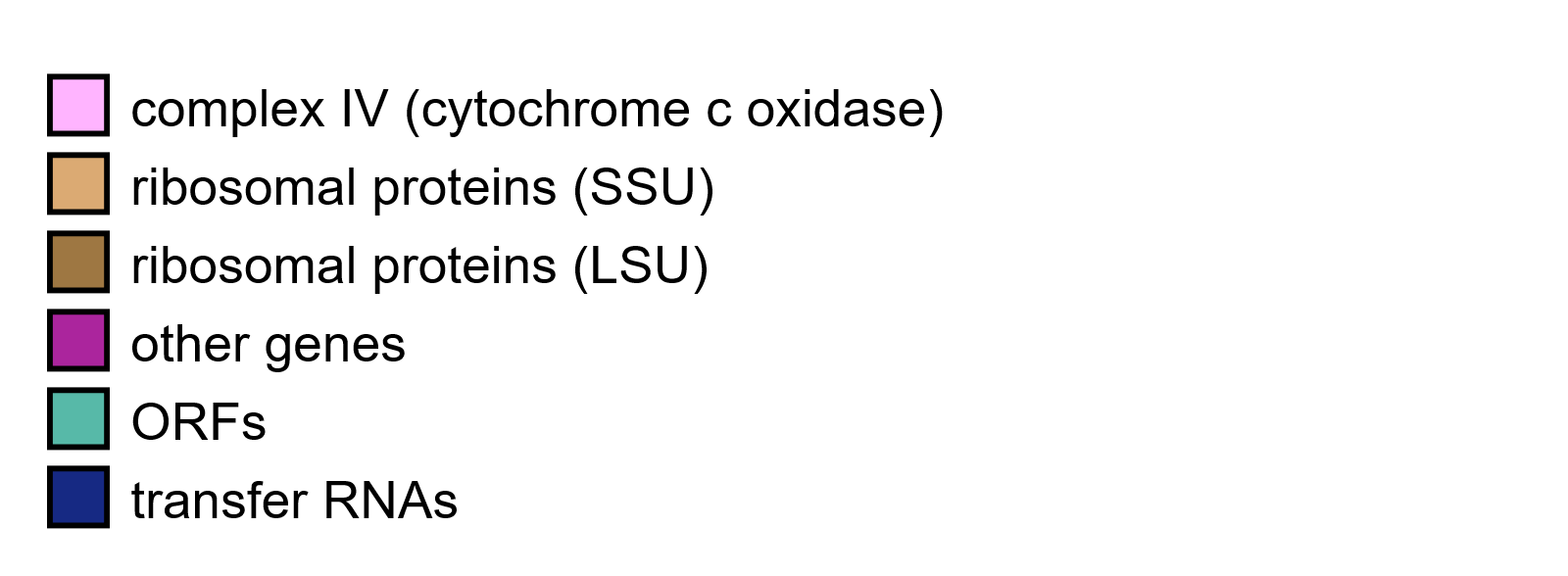


**Figure S1**. Map of somatic hybrid mtDNA. **c.** SH9A, chromosome 3, 49622 bp, GenBank acc.no: ON682439

**d.**


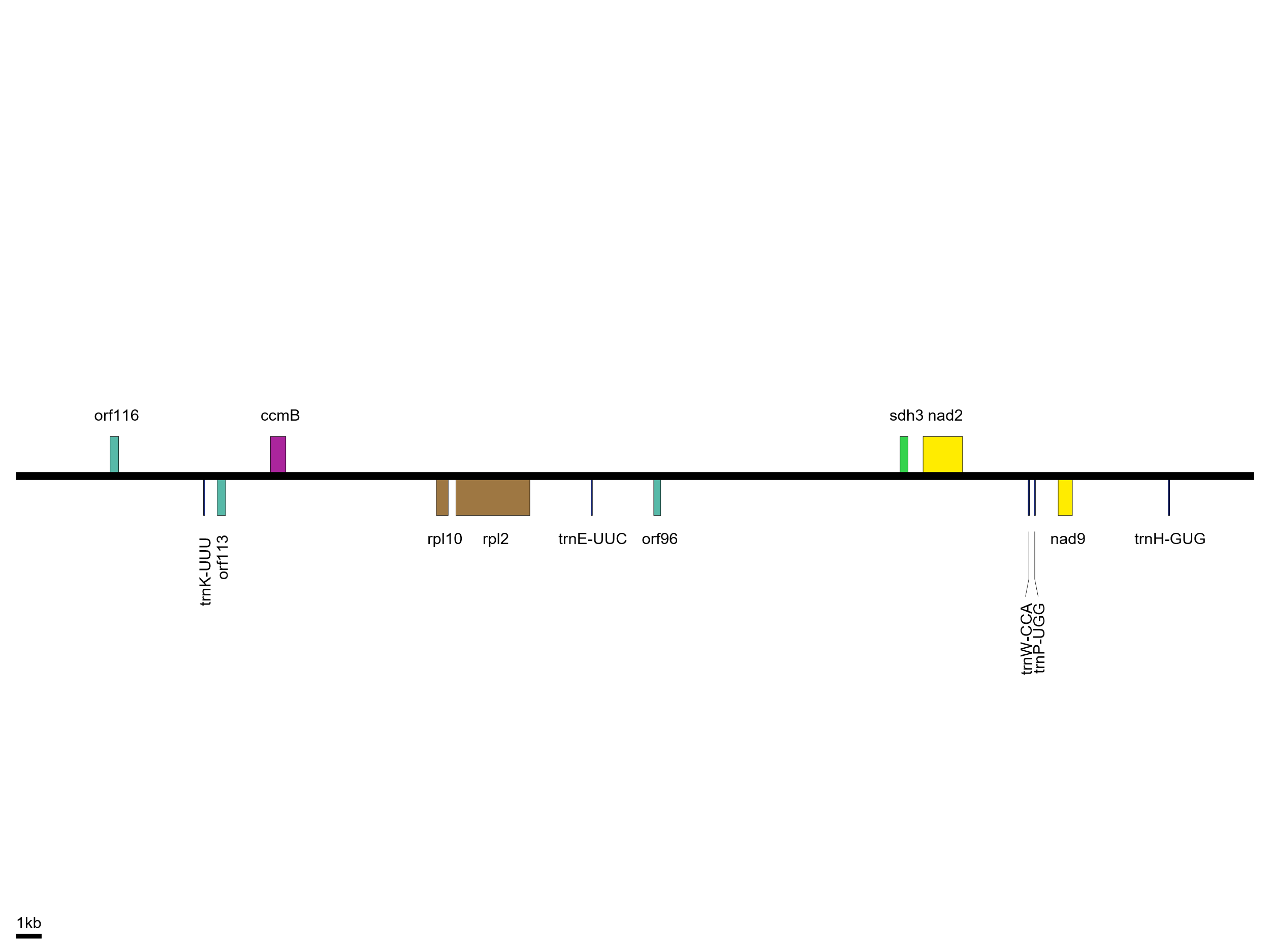


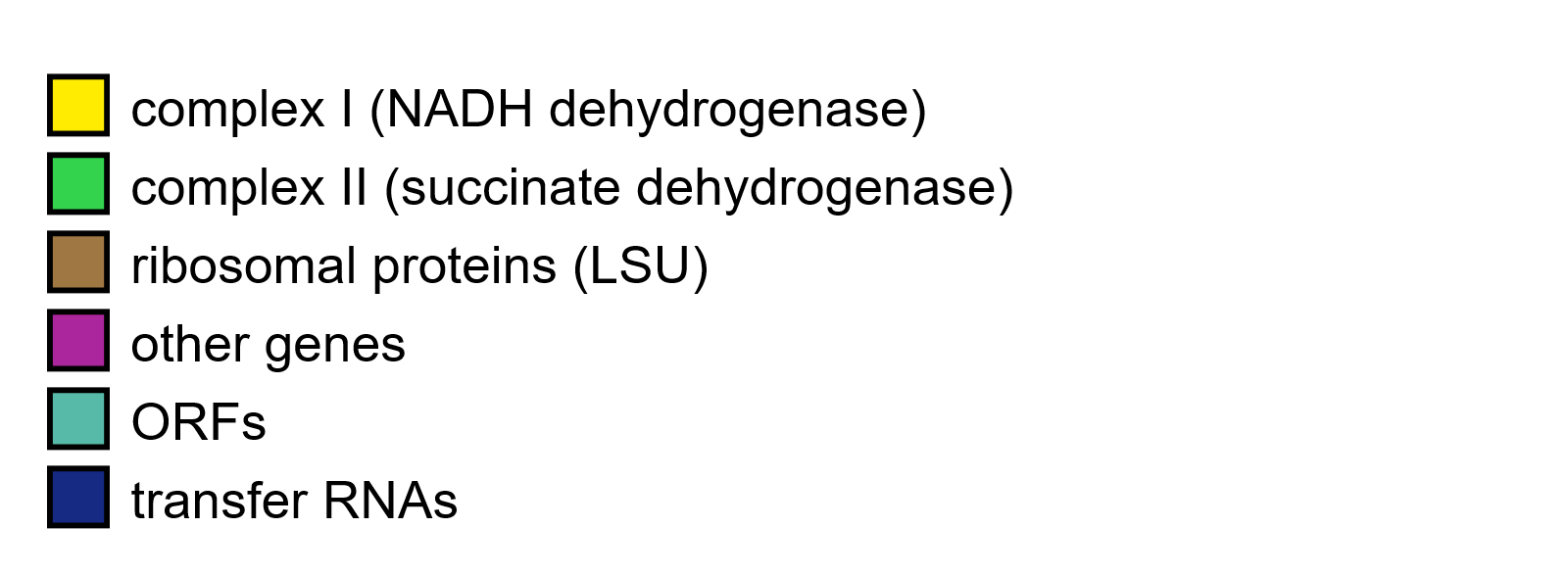


**Figure S1**. Map of somatic hybrid mtDNA. **d.** SH9A, chromosome 4, 48445 bp, GenBank acc.no: ON682440


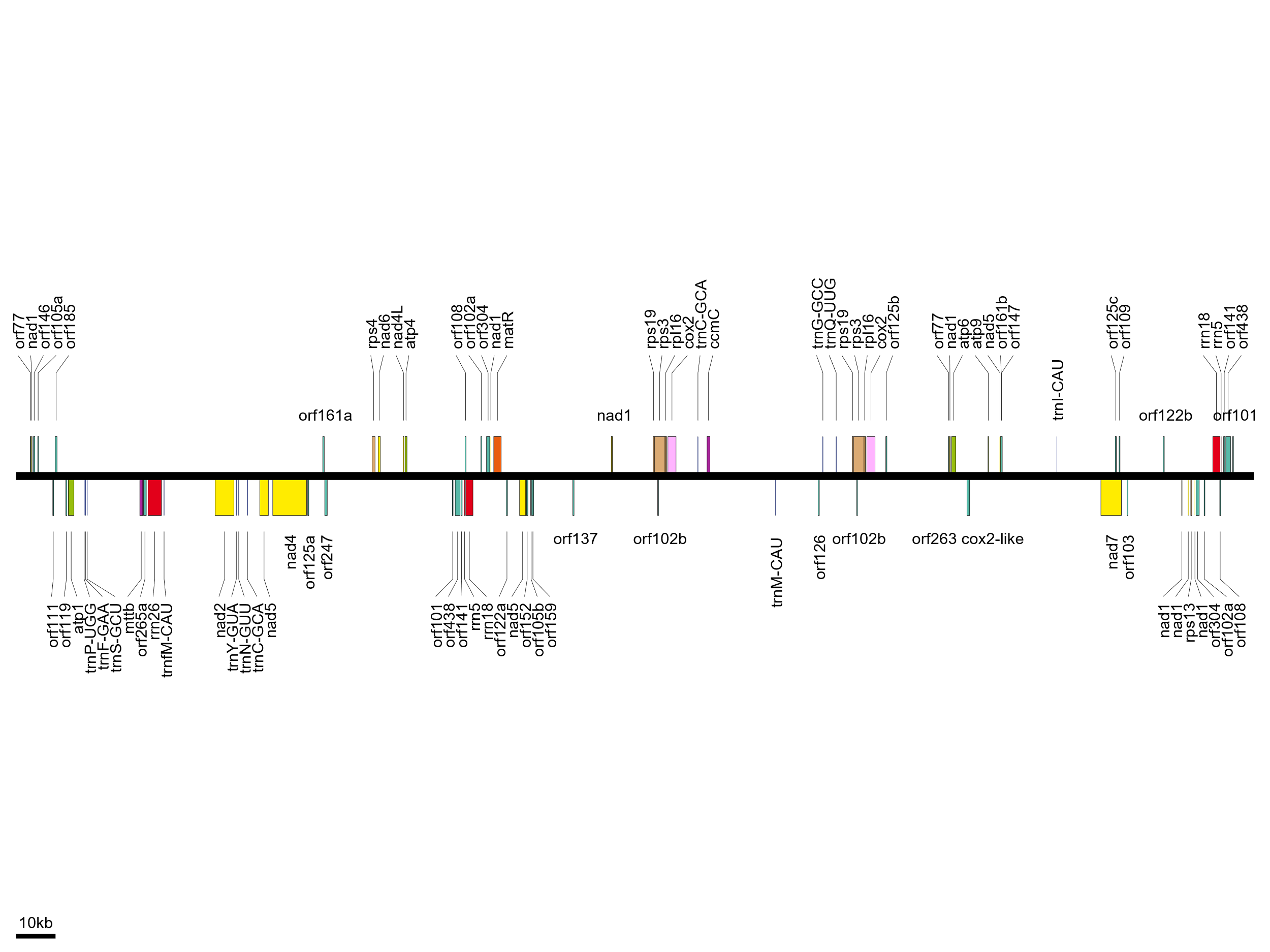
**e.**

**
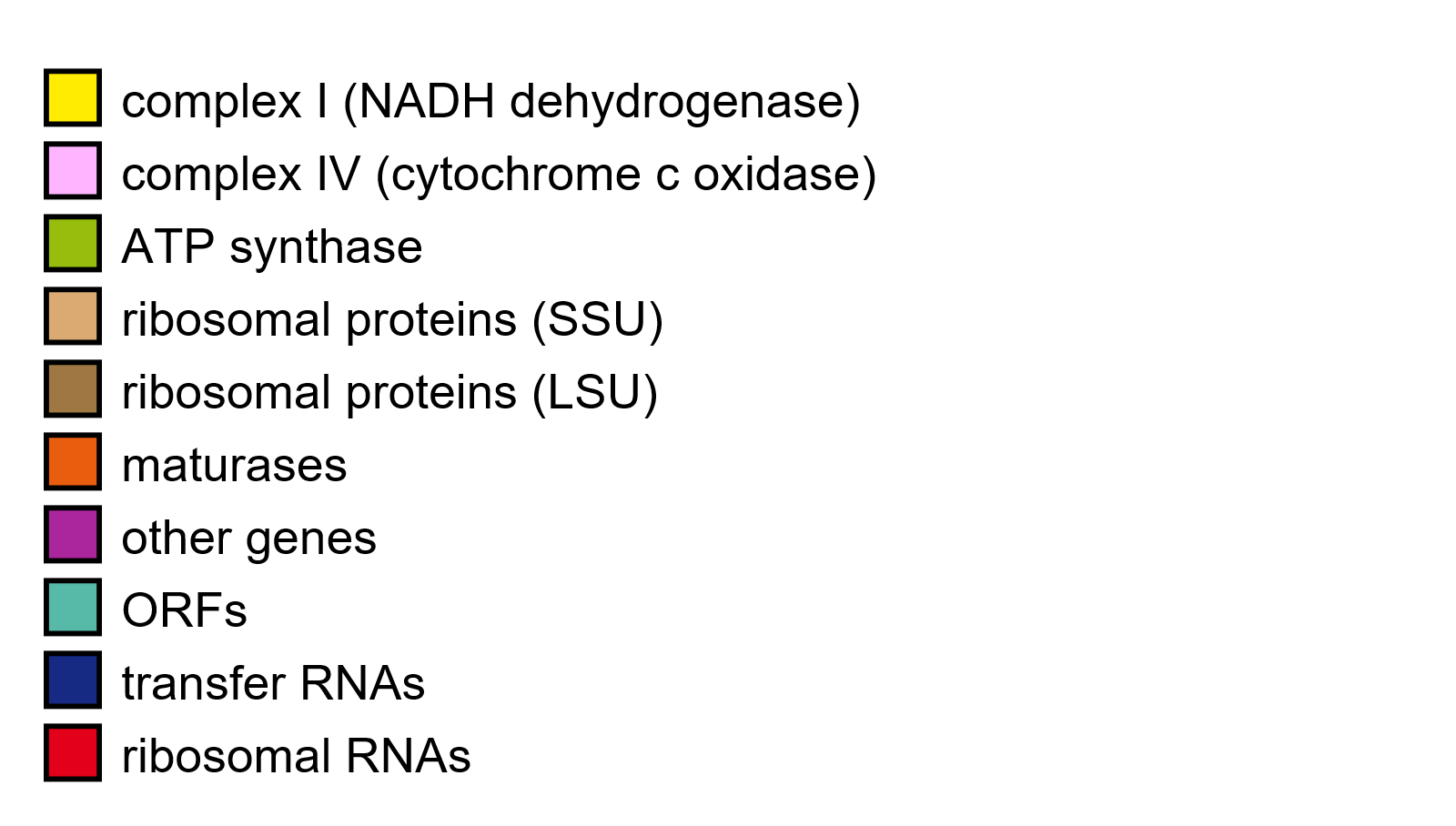
**

**Figure S1**. Map of somatic hybrid mtDNA. **e.** SH9B, chromosome 1, 313767 bp, GenBank acc.no: ON009139


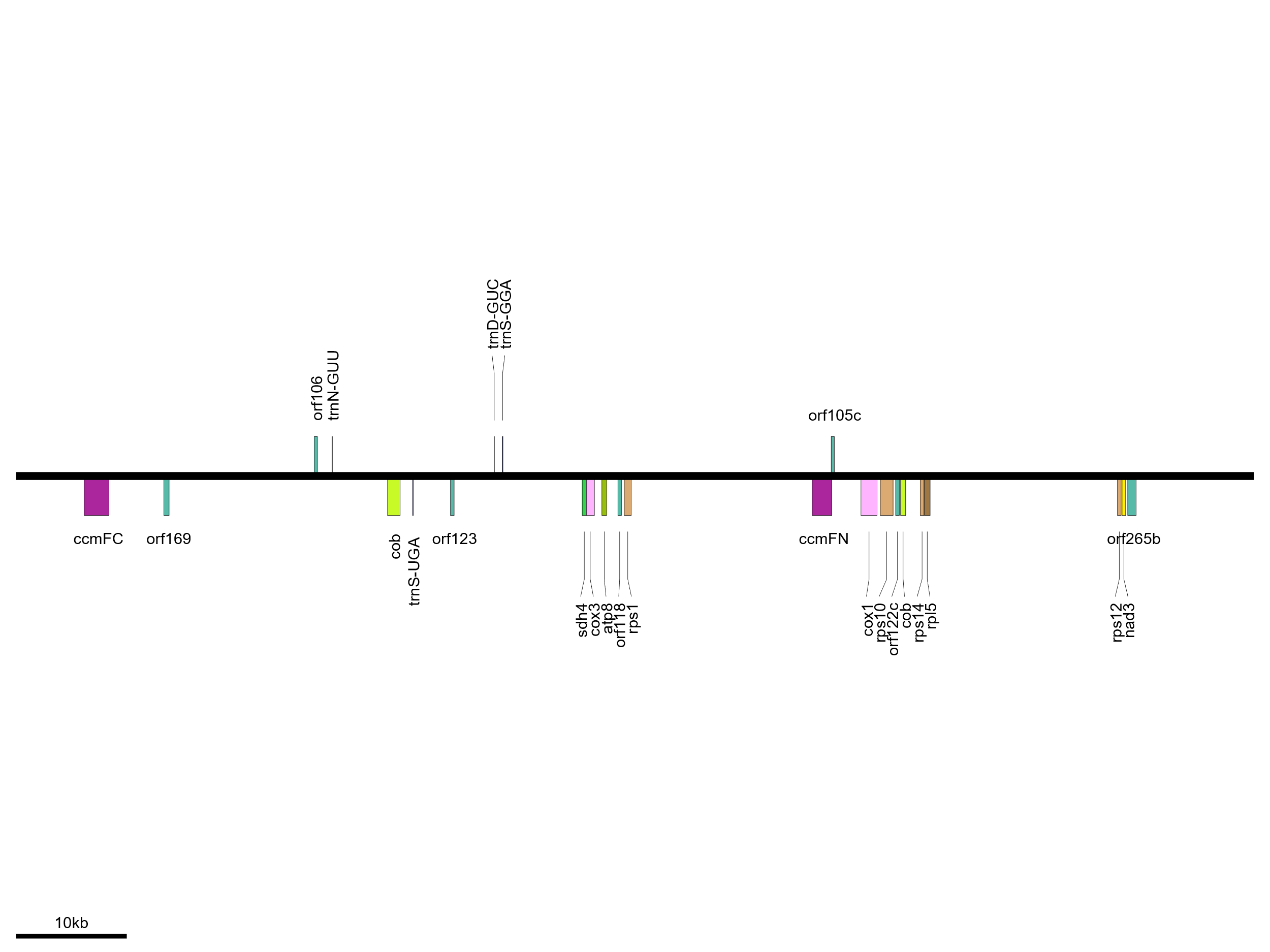
**f.**

**
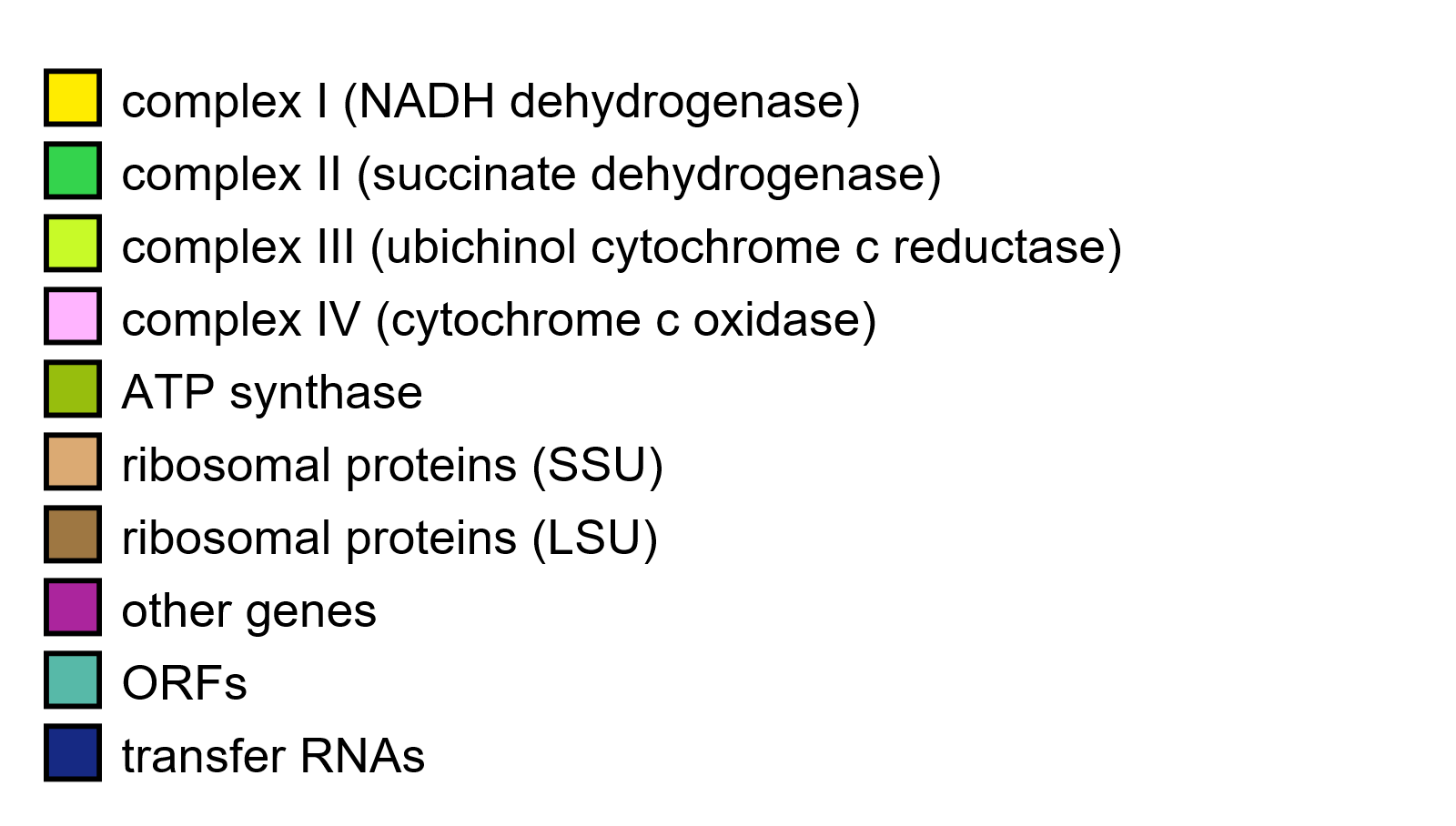
**

**Figure S1**. Map of somatic hybrid mtDNA. **f.** SH9B, chromosome 2, 111810 bp, GenBank acc.no: ON009140

**g.**


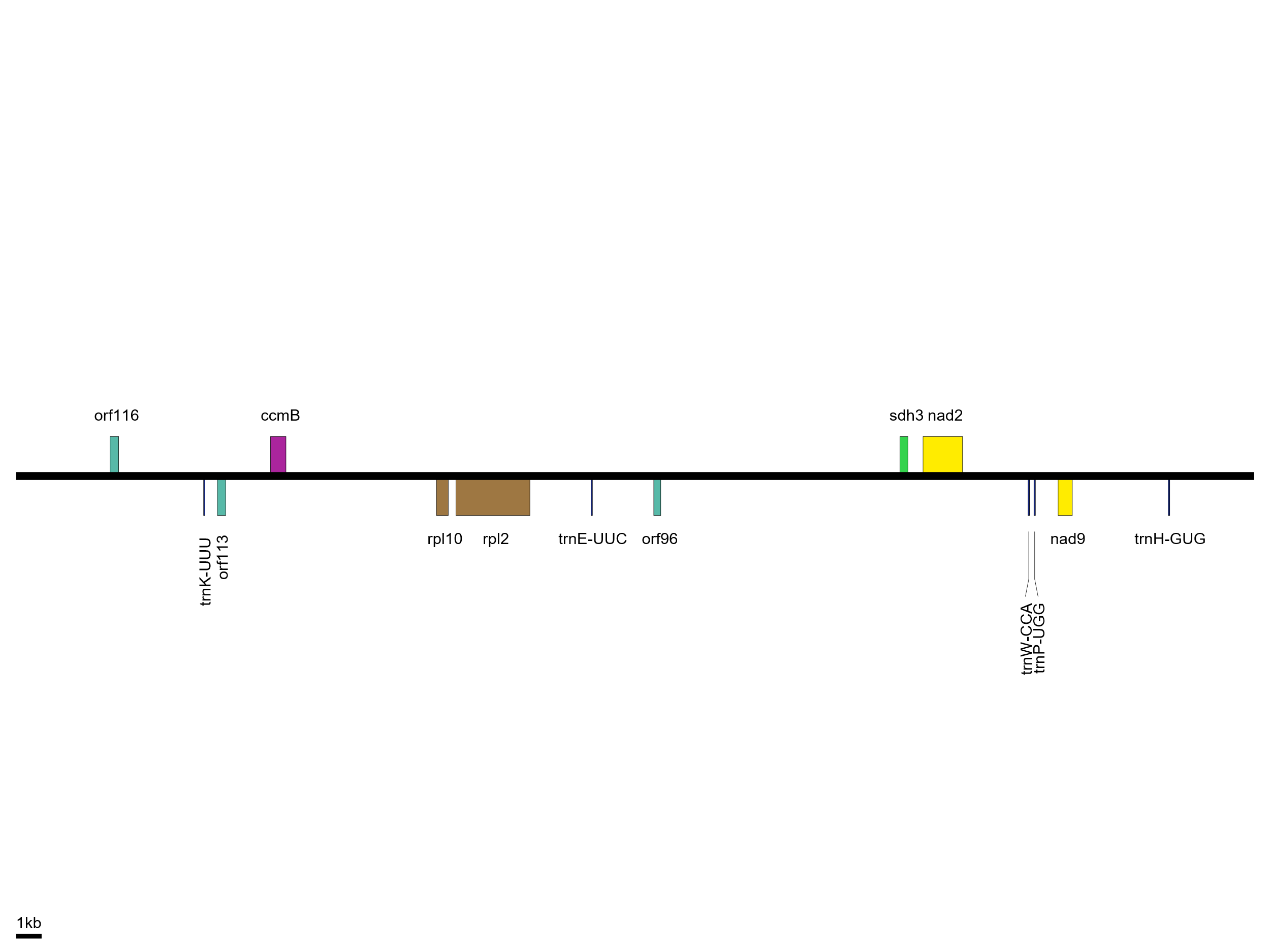


**
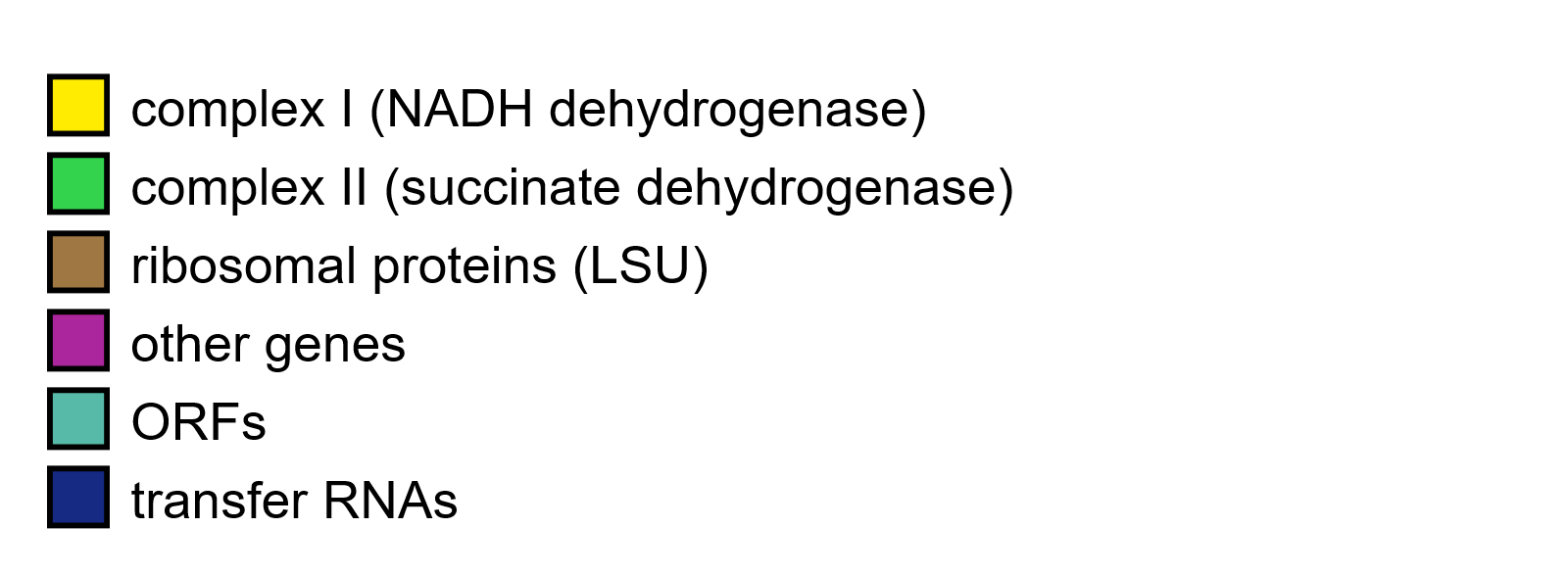
**

**Figure S1**. Map of somatic hybrid mtDNA. **g.** SH9B, chromosome 3, 48452 bp, GenBank acc.no: ON009141
