# Supplementary material for: Mitochondrial gene editing and allotopic expression unveil the role of orf125 in the induction of male fertility in some Solanum spp. hybrids and in the evolution of the common potato
Source: Plant Biotechnol J. 2025 Mar 22;23(5):1862–75. doi: 10.1111/pbi.70012 (PMC12018842; doi:10.1111/pbi.70012)
Supplement: Supplementary file 2 — Figure S2 Nucleotide sequence of orf125 and deduced protein sequence in SH9B. [file PBI-23-1862-s006.docx]

**Figure S2.** Nucleotide sequence of *orf125* and deduced protein sequence in SH9B. Start and stop codons are indicated in bold, while the dotted line indicates the predicted transmembrane domain.
