# Supplementary material for: Mitochondrial gene editing and allotopic expression unveil the role of orf125 in the induction of male fertility in some Solanum spp. hybrids and in the evolution of the common potato
Source: Plant Biotechnol J. 2025 Mar 22;23(5):1862–75. doi: 10.1111/pbi.70012 (PMC12018842; doi:10.1111/pbi.70012)
Supplement: Supplementary file 3 — Figure S3 Schematic representation and description of vectors used for overexpression of orf125 CMS‐candidate gene in the male‐fertile somatic hybrid SH9A. [file PBI-23-1862-s014.docx]

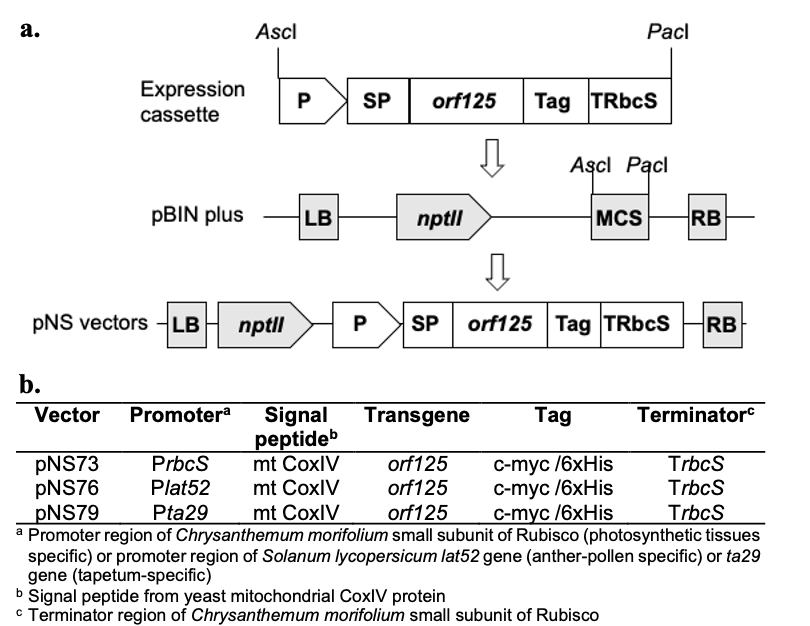


**Figure S3.** Schematic representation (**a.**) and description (**b.**) of vectors used for overexpression of *orf125* CMS-candidate gene in the male fertile somatic hybrid SH9A.
