# Supplementary material for: Mitochondrial gene editing and allotopic expression unveil the role of orf125 in the induction of male fertility in some Solanum spp. hybrids and in the evolution of the common potato
Source: Plant Biotechnol J. 2025 Mar 22;23(5):1862–75. doi: 10.1111/pbi.70012 (PMC12018842; doi:10.1111/pbi.70012)
Supplement: Supplementary file 4 — Figure S4 Selection of positive orf125 transgenic plants after transformation with pNS73, pNS76 and pNS79 vectors. [file PBI-23-1862-s011.docx]

Figure S4. Selection of positive *orf125* transgenic plants after transformation with pNS73, pNS76 and pNS79 vectors. The transgene fragment was amplified with P*rbcS* F/*orf125* *Bgl*II R, P*lat52* F/*orf125* *Bgl*II R and P*ta29* F/*orf125* *Bgl*II R primers, respectively (see Table S5). 9A, SH9A; C+, DNA vectors.
