# Supplementary material for: Mitochondrial gene editing and allotopic expression unveil the role of orf125 in the induction of male fertility in some Solanum spp. hybrids and in the evolution of the common potato
Source: Plant Biotechnol J. 2025 Mar 22;23(5):1862–75. doi: 10.1111/pbi.70012 (PMC12018842; doi:10.1111/pbi.70012)
Supplement: Supplementary file 5 — Figure S5 Male fertility of SH9A transgenic plants expressing orf125 under the control of PrbcS (NS73), Plat52 (NS76) and Pta29 (NS79) promoters. [file PBI-23-1862-s003.docx]

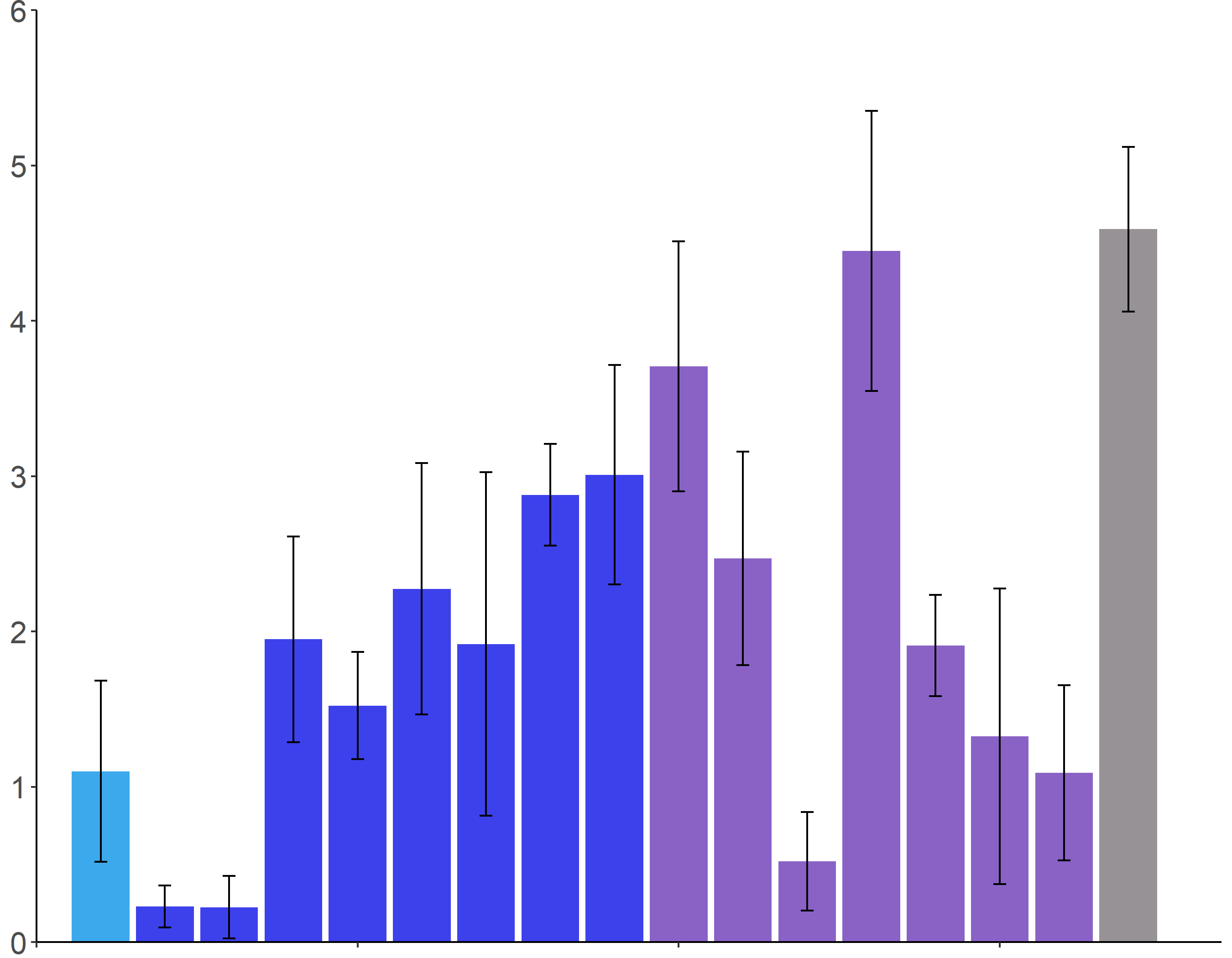


**Pollen production (mg)**

NS73

NS76

NS79

1C

SH9A

2A

5A

6B

8A

10B

15A

19A

1A

2A

7A

8A

9A

10A

16A

18A

SH9B

50

33

35

72

73

68

74

71

63

74

53

65

65

57

44

68

100

Figure S5. Male fertility of SH9A transgenic plants expressing *orf125* under the control of P*rbcS* (NS73), P*lat52* (NS76) and P*ta29* (NS79) promoters. Bars represent pollen production (mg/flower + SD). Numbers above bars indicate average pollen stainability (%).
