# Supplementary material for: Mitochondrial gene editing and allotopic expression unveil the role of orf125 in the induction of male fertility in some Solanum spp. hybrids and in the evolution of the common potato
Source: Plant Biotechnol J. 2025 Mar 22;23(5):1862–75. doi: 10.1111/pbi.70012 (PMC12018842; doi:10.1111/pbi.70012)
Supplement: Supplementary file 6 — Figure S6 Effects of the expression of gus gene on pollen production and stainability. [file PBI-23-1862-s008.docx]

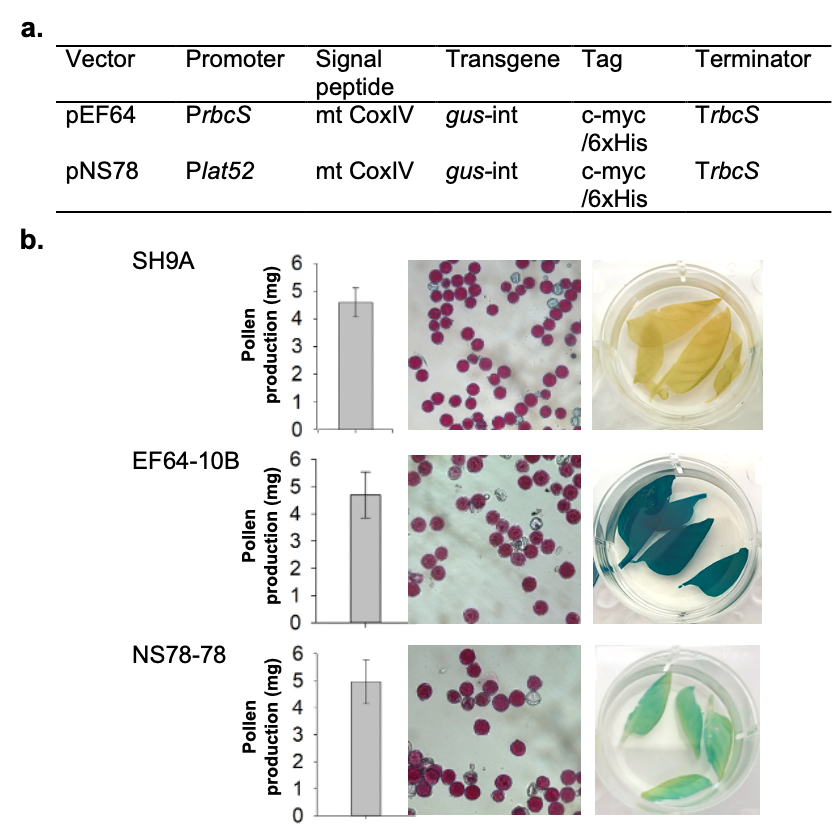


**Figure S6.** Effects of the expression of *gus* gene on pollen production and stainability. **a.** Description of vectors used for overexpression of the *gus* gene. **b.** Pollen production and stainability, and results of histochemical GUS assay in leaves of the male fertile somatic hybrid SH9A and of selected transgenic plants (EF64-10B and NS78-78).
