# Supplementary material for: Mitochondrial gene editing and allotopic expression unveil the role of orf125 in the induction of male fertility in some Solanum spp. hybrids and in the evolution of the common potato
Source: Plant Biotechnol J. 2025 Mar 22;23(5):1862–75. doi: 10.1111/pbi.70012 (PMC12018842; doi:10.1111/pbi.70012)
Supplement: Supplementary file 7 — Figure S7 Putative groups of accessions obtained by BLAST analysis with the SH9B orf247‐nad4 sequence as query and Neighbour Joining Method. [file PBI-23-1862-s013.docx]

Figure S7. Putative groups of accessions obtained by BLAST analysis with the SH9B *orf247-nad4* sequence (P11-P3 primers, see Table S5) as query and Neighbor Joining Method.
