# Supplementary material for: Mitochondrial gene editing and allotopic expression unveil the role of orf125 in the induction of male fertility in some Solanum spp. hybrids and in the evolution of the common potato
Source: Plant Biotechnol J. 2025 Mar 22;23(5):1862–75. doi: 10.1111/pbi.70012 (PMC12018842; doi:10.1111/pbi.70012)
Supplement: Supplementary file 8 — Figure S8 Dot Plots between the sequence of the “orf247‐nad4” SH9B region and corresponding sequences identified in some GenBank accessions. [file PBI-23-1862-s010.docx]

Figure S8. Dot Plots between the sequence of the “*orf247-nad4*” SH9B region (corresponding to the fragment between nucleotides 78705 and 73605 in ON009139, amplified by P11-P3 primers and including *orf125*, see Table S5) and corresponding sequences identified in some GenBank accessions. The x-and y-axes report the SH9B and corresponding sequences, respectively. Plus/plus matches are in blue, whereas plus/minus matches are in orange.
