# Supplementary material for: Mitochondrial gene editing and allotopic expression unveil the role of orf125 in the induction of male fertility in some Solanum spp. hybrids and in the evolution of the common potato
Source: Plant Biotechnol J. 2025 Mar 22;23(5):1862–75. doi: 10.1111/pbi.70012 (PMC12018842; doi:10.1111/pbi.70012)
Supplement: Supplementary file 9 — Figure S9 Models of ORF125 from S. tuberosum Group Tuberosum (=SH9B) obtained by various predictors and of mutant forms by AlphaFold. [file PBI-23-1862-s005.docx]

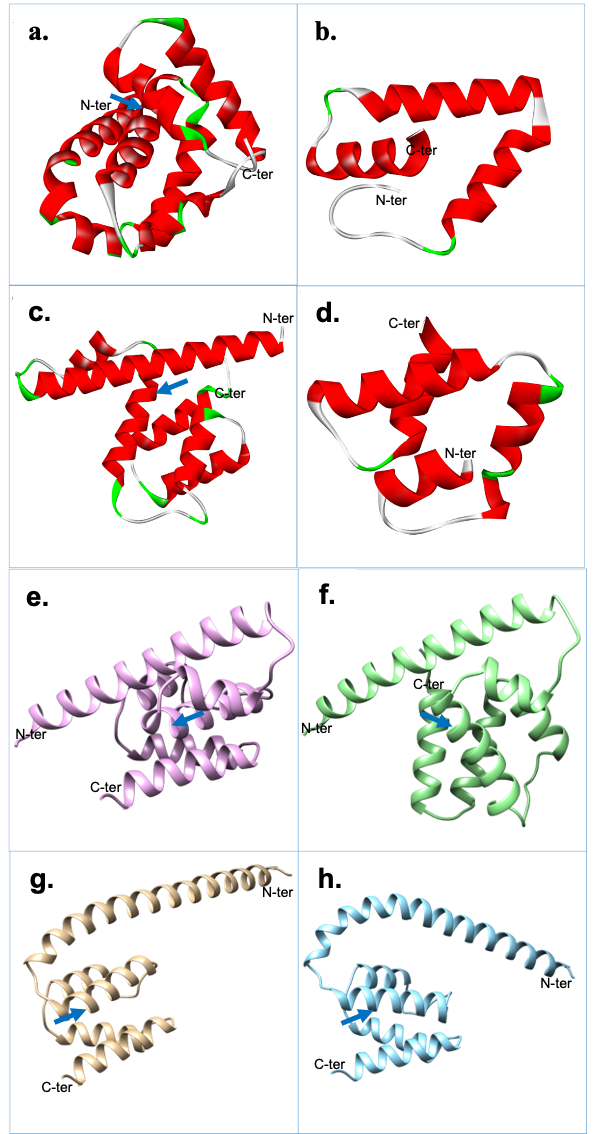


**Figure S9.** Models of ORF125 from *S. tuberosum* Group *Tuberosum* (= SH9B) obtained by various predictors (a–e) and of mutant forms by AlphaFold (f-h). Position 55 is pointed by the blue arrow position, when included in the model. **a.** Model obtained by I-TASSER of the complete sequence; it is the first in the top ten models produced by the predictor and shows a Z-score equal to -5.22. **b.** The best model obtained by SWISS MODEL; it covers only a portion of the sequence from Leu64 to Lys119 and shows a Z-score equal to -5.9. **c.** Complete model of the protein obtained by AlphaFold with a Z-score of -4.72. **d.** Model obtained by Phyre2 of the ORF125 region from Ile59 to Trp121; it is at the eighth place in the top ten models produced by the predictor but reaches the best Z-score, with a value equal to -6.28. **e.** Same as **c.**: in this case model is rotated by almost 180 degrees to allow a better comparison to the models presented in the subsequent panels. **f.** The best model obtained for the edited ORF125 with the D55N mutation, the model shows a Z-score equal to -3.87. **g.** The complete model of the protein ORF125 from *S. tuberosum* Group *Andigenum* (*adg1)* with a Z-score of -3.46. **h.** Model of ORF125 from *S. wrightii/sisymbriifolium/torvum;* it reaches a Z-score with a value equal to -3.46.
