# Supplementary material for: Mitochondrial gene editing and allotopic expression unveil the role of orf125 in the induction of male fertility in some Solanum spp. hybrids and in the evolution of the common potato
Source: Plant Biotechnol J. 2025 Mar 22;23(5):1862–75. doi: 10.1111/pbi.70012 (PMC12018842; doi:10.1111/pbi.70012)
Supplement: Supplementary file 10 — Figure S10 Alignments of ORF125 from SH9B (ON009139)/cv. Désirée (MN104801), edited SH9B, S. tuberosum Group Andigenum (MW122969), S. wrightii (MT122958), S. sisymbriifolium (MT122964), S. torvum (MT122979). [file PBI-23-1862-s002.docx]

**Figure S10.** Alignments of ORF125 from SH9B (ON009139) / *cv.* Désirée (MN104801), edited SH9B (Nicolia et al., 2024), *S. tuberosum* Group *Andigenum* (MW122969), *S. wrightii* (MT122958), *S. sisymbriifolium* (MT122964), *S. torvum* (MT122979).

**References**

Nicolia A, Scotti N, D’Agostino N, Festa G, Sannino L, Aufiero G, Arimura S, Cardi T (2024) Mitochondrial DNA editing in potato through mitoTALEN and mitoTALECD: molecular characterization and stability of editing events. *Plant Methods* **20**: 4.
