# Supplementary material for: Mitochondrial gene editing and allotopic expression unveil the role of orf125 in the induction of male fertility in some Solanum spp. hybrids and in the evolution of the common potato
Source: Plant Biotechnol J. 2025 Mar 22;23(5):1862–75. doi: 10.1111/pbi.70012 (PMC12018842; doi:10.1111/pbi.70012)
Supplement: Supplementary file 11 — Figure S11 Root Mean Square Deviation (RMSD) values derived by the comparison of wt (tbr) and mutant forms of ORF125. [file PBI-23-1862-s012.docx]

**Figure S11.** Root Mean Square Deviation (RMSD) values derived by the comparison of wt (*tbr*) and mutant forms of the entire ORF125 protein or the N and C-terminal regions (from aminoacids 1 to 41 and from 42 to 125, respectively).
