# Supplementary material for: Mitochondrial gene editing and allotopic expression unveil the role of orf125 in the induction of male fertility in some Solanum spp. hybrids and in the evolution of the common potato
Source: Plant Biotechnol J. 2025 Mar 22;23(5):1862–75. doi: 10.1111/pbi.70012 (PMC12018842; doi:10.1111/pbi.70012)
Supplement: Supplementary file 12 — Figure S12 Cytoplasm distribution in Andean, Chilean and European cultivated tetraploid potato accessions and putative role of orf125 in common potato evolution. [file PBI-23-1862-s016.docx]

Figure S12. Cytoplasm distribution in Andean, Chilean and European cultivated tetraploid potato accessions and putative role of *orf125* in common potato evolution. Within the *berthaultii* complex in South Bolivia and North Argentina (inset), accessions crossed as female with tetraploid *Andigenum* species contributing not only the T-type plastomes but also the chondriomes containing the CMS-inducing *orf125*. This genetic exchange led to the emergence of the new species within the *Chilotanum* group. The novel T/β-cytoplasm subsequently spread to Europe and other parts of the world. Its prevalence in modern cultivars across various collections varies, depending on the use of wild species in breeding programs (Hosaka and Sanetomo, 2012). Data of the *Andigenum* and *Chilotanum* Groups are sourced from Hosaka and Hanneman (1988) (with European and American cultivars omitted), while information on European cultivars is from Sanetomo and Gebhardt (2015). Modified from Peralta et al. (2021) and Spooner et al. (2007).

**References**

Hosaka K, Hanneman RE (1988) The origin of the cultivated tetraploid potato based on chloroplast DNA. *Theoretical and Applied Genetics* **76**: 172–176.

Hosaka K, Sanetomo R (2012) Development of a rapid identification method for potato cytoplasm and its use for evaluating Japanese collections. *Theoretical and Applied Genetics* **125**: 1237–1251.

Peralta IE, Clausen AM, Zorrilla C, Ames M, Digilio A, Rodriguez F (2021) Wild and Cultivated Potato Species Diversity, Taxonomy, and Conservation. In: *The Wild Solanums Genomes. Compendium of Plant Genomes* (Carputo D, Aversano R, Ercolano MR, eds), pp 51–94. Cham: Springer.

Sanetomo R, Gebhardt C (2015) Cytoplasmic genome types of European potatoes and their effects on complex agronomic traits. *BMC Plant Biol* **15**: 162.

Spooner DM, Fajardo D, Bryan GJ (2007) Species limits of *Solanum berthaultii* Hawkes and *S. tarijense* Hawkes and the implications for species boundaries in *Solanum* sect. *Petota*. *Taxon* **56**: 987–999.
