# Supplementary material for: Mitochondrial gene editing and allotopic expression unveil the role of orf125 in the induction of male fertility in some Solanum spp. hybrids and in the evolution of the common potato
Source: Plant Biotechnol J. 2025 Mar 22;23(5):1862–75. doi: 10.1111/pbi.70012 (PMC12018842; doi:10.1111/pbi.70012)
Supplement: Supplementary file 13 — Table S1 Comparison of the mitochondrial genomes of the somatic hybrids SH9A and SH9B. [file PBI-23-1862-s007.docx]

**Table S1.** Comparison of the mitochondrial genomes of the somatic hybrids SH9A and SH9B.

|  | SH9A | | | |  | SH9B | | |
| --- | --- | --- | --- | --- | --- | --- | --- | --- |
| Chromosomes | 1 | 2 | 3 | 4 |  | 1 | 2 | 3 |
| GenBank acc. no. | ON682437 | ON682438 | ON682439 | ON682440 |  | ON009139 | ON009140 | ON009141 |
| Genome length (bp) | 251363 | 109928 | 49622 | 48445 |  | 313767 | 111810 | 48452 |
| Total genes^a^ | 94 (109) | | | |  | 101 (118) | | |
| Protein coding genes^a^ | 37 (41) | | | |  | 37 (41) | | |
| Pseudogenes | 1 | | | |  |  | 2 |  |
| Hypothetical genes^a^ | 35 (41) | | | |  | 41 (49) | | |
| rRNA^a^ | 3 (5) | | | |  | 3 (5) | | |
| tRNA^a^ | 18 (21) | | | |  | 18 (21) | | |
| Total genes^ab^ | 55 (62) | 22 (23) | 5 (11) | 12 (13) |  | 65 (80) | 24 (25) | 12 (13) |
| Protein coding genes^ab^ | 19 | 12 | 1 (5) | 5 |  | 20 (24) | 12 | 5 |
| Pseudogenes^b^ | 0 | 1 | 0 | 0 |  | 0 | 2 | 0 |
| Hypothetical genes^ab^ | 25 (30) | 6 | 1 (2) | 3 |  | 31 (39) | 7 | 3 |
| rRNA^ab^ | 3 (5) | 0 | 0 | 0 |  | 3 (5) | 0 | 0 |
| tRNA^ab^ | 8 | 3 (4) | 3 (4) | 4 (5) |  | 11 (12) | 3 (4) | 4 (5) |
| GC%^b^ | 45.40 | 44.56 | 44.58 | 44.59 |  | 45.19 | 44.59 | 44.59 |
| AT/GC^b^ | 1.20 | 1.24 | 1.24 | 1.24 |  | 1.21 | 1.24 | 1.24 |

^a^ First value excludes duplicated; value in parentheses includes them.

^b^ Values referring to individual chromosomes
