# Supplementary material for: Mitochondrial gene editing and allotopic expression unveil the role of orf125 in the induction of male fertility in some Solanum spp. hybrids and in the evolution of the common potato
Source: Plant Biotechnol J. 2025 Mar 22;23(5):1862–75. doi: 10.1111/pbi.70012 (PMC12018842; doi:10.1111/pbi.70012)
Supplement: Supplementary file 16 — Table S4 List of Solanum spp. genotypes used in this study. [file PBI-23-1862-s009.docx]

**Table S4.** List of *Solanum* spp. genotypes used in this study.

| Species | Accession**^a^** | Code | Origin | Ploidy | Notes |
| --- | --- | --- | --- | --- | --- |
| *S. berthaultii* | PI 498075 | *ber1* | Bolivia | 2*x* |  |
|  | PI 498095 | *ber2* | Bolivia | 2*x* |  |
|  | PI 498101 | *ber3* | Bolivia | 2*x* |  |
| *S. brachistotrichum* | PI 320265 | *bst* | Mexico | 2*x* |  |
| *S. bulbocastanum* | PI 275187 | *blb* | Mexico | 2*x* |  |
| S. cardiophyllum | PI 347759 | *cph* | Mexico | 2*x* |  |
| *S. chacoense* | PI 320282 | *chc* | Argentina | 2*x* |  |
| *S. commersonii* | PI 243503 | *cmm* | Argentina | 2*x* |  |
| *S. etuberosum* | PI 558054 | *etb* | Chile | 2*x* |  |
| *S. infundibuliforme* | PI 472857 | *ifd* | Argentina | 2*x* |  |
| *S. nigrum* | - | *ngr* | - | 6*x* |  |
| *S. pinnatisectum* | PI 275236 | *pnt* | Mexico | 2*x* |  |
| *S. polytrichon* | PI 184773 | *plt* | Mexico | 4*x* |  |
| *S. raphanifolium* | PI 265878 | *rap* | Peru | 2*x* |  |
| *S. sanctae-rosae* | PI 218221 | *sct* | Argentina | 2*x* |  |
| *S. sparsipilum* | PI 234014 | *spl2* | Bolivia | 2*x* |  |
| *S. spegazzinii* | PI 205394 | *spg* | Argentina | 2*x* |  |
| *S. tarijense* | PI 265577 | *tar1* | Bolivia | 2*x* |  |
|  | PI 414148 | *tar2* | Argentina | 2*x* |  |
|  | PI 442689 | *tar3* | Argentina | 2*x* |  |
| *S. trifidum* | PI 255541 | *trf* | Mexico | 2*x* |  |
| *S. tuberosum* Group *Andigenum* | CPC 843 | *adg* | - | 4*x* |  |
| *S. tuberosum* Group *Tuberosum* | - | Des | - | 4*x* | *cv.* Désirée (Urgenta x Depesche)^b^ |
|  | DH81-7-1463 | SVP11 | - | 2*x* | Dihaploid clone of W72-22-492 |
| *cmm* (+) SVP11^c^ | - | SH1A | - | 4*x* | Somatic hybrid, male sterile |
| *cmm* (+) SVP11 | - | SH7A | - | 4*x* | Somatic hybrid, male sterile |
| *cmm* (+) SVP11 | - | SH9A | - | 4*x* | Somatic hybrid, male fertile |
| *cmm* (+) SVP11 | - | SH9B | - | 4*x* | Somatic hybrid, male sterile |
| *cmm* (+) SVP11 | - | SH12A | - | 4*x* | Somatic hybrid, male sterile |
| *cmm* (+) SVP11 | - | SH25A | - | 6*x* | Somatic hybrid, male sterile |

^a^ PI and CPC accessions were kindly provided by Potato Introduction Station, Sturgeon Bay, Wisconsin, and Commonwealth Potato Collection, Dundee, UK, respectively; - = not available/relevant

^b^ <https://www.plantbreeding.wur.nl/PotatoPedigree/index.html>

^c^ Cardi et al. (1993)

**References**

Cardi T, D’Ambrosio F, Consoli D, Puite KJ, and Ramulu KS (1993) Production of somatic hybrids between frost-tolerant *Solanum commersonii* and *S. tuberosum*: characterization of hybrid plants. *Theoretical and Applied Genetics* **87**: 193–200.
