# Supplementary material for: Mitochondrial gene editing and allotopic expression unveil the role of orf125 in the induction of male fertility in some Solanum spp. hybrids and in the evolution of the common potato
Source: Plant Biotechnol J. 2025 Mar 22;23(5):1862–75. doi: 10.1111/pbi.70012 (PMC12018842; doi:10.1111/pbi.70012)
Supplement: Supplementary file 17 — Table S5 List of primers used. [file PBI-23-1862-s004.docx]

**Table S5.** List of primers used.

| **Primers** | **Sequence (5’-3’)** | **Tm (°C)** | **Use** |
| --- | --- | --- | --- |
| orf125 NcoI F | CATGCCATGGGCATGAATATCTTTGATATTTTC | 76 | *orf125* cloning in plant vectors |
| orf125 BglII R | CTAGATCTGCTAGAGGAAAGGTCCAATCTT | 77 | *orf125* cloning in plant vectors / PCR analysis of transgenic plants |
| P*rbcS* F | CCGTTAGATAGCAAACAACA | 64 | PCR analysis of transgenic plants |
| P*lat52* F | AGGCGCGCCCCTATACCCCTTGGATAA | 81 | *orf125* cloning in plant vectors / PCR analysis of transgenic plants |
| P*lat52* R | CTCTAGATTTAAATTGGAATTTTTTTTTTTGG | 69 | *orf125* cloning in plant vectors / PCR analysis of transgenic plants |
| P*ta29* F | AGGCGCGCCAACTGGTCTCAACCTCGTA | 81 | *orf125* cloning in plant vectors / PCR analysis of transgenic plants |
| P*ta29* R | CTCTAGATTTTAGCTAAGTTTATTTAAG | 69 | *orf125* cloning in plant vectors / PCR analysis of transgenic plants |
| RT orf125 F | CGTAGCCAGACACAAACTTTC | 69 | RT-PCR analysis of *orf125* expression in somatic hybrids |
| RT orf125 R | TGCAAAGCCATCAAGACCCA | 68 | RT-PCR analysis of *orf125* expression in somatic hybrids |
| RT orf125-nad4F | CCGAAGAACAGGGGGATACA | 64 | RT-PCR analysis of *orf125-nad4* co-expression |
| RT orf125-nad4R | ATACCTTCGGCTGCTTGTCC | 64 | RT-PCR analysis of *orf125-nad4* co-expression |
| RT 18S F | TAGATAAAAGGTCGACGCGG | 68 | RT-PCR analysis of *rrn18* expression in somatic hybrids |
| RT 18S R | CCCAAAGTCCAACTACGAGC | 70 | RT-PCR analysis of *rrn18* expression in somatic hybrids |
| qRT-PCR orf125 F | CCACAAAATTCACGAGGGCT | 68 | qRT-PCR analysis of *orf125* expression in transgenic plants |
| qRT-PCR orf125 R | AGCCATCAAGACCCATTCACT | 69 | qRT-PCR analysis of *orf125* expression in transgenic plants |
| qRT-PCR ef1α F | ATTGGAAACGGATATGCTCCA | 63 | qRT-PCR analysis of *ef1α* expression in transgenic plants |
| qRT-PCR ef1α R | TCCTTACCTGAACGCCTGTCA | 66 | qRT-PCR analysis of *ef1α* expression in transgenic plants |
| P4 | TTATAGAGGAAAGGTCCAATCTTICA | 62 | PCR amplification of *orf125* coding sequence |
| P5 | ATGAATATCTTTGATATTTTCACG | 63 | PCR amplification of *orf125* coding sequence |
| P3 | GCACGGGACAAAGAATAAGACC | 62 | PCR amplification of *orf247-nad4* genomic region |
| P11 | TTATTATTCGGGCGGGGCTC | 60 | PCR amplification of *orf247-nad4* genomic region |
